# miRNA Profiling Reveals the Role of Gibberellin Signaling Pathway in Low-Nitrogen Stress Adaptation of Xinjiang Spring Wheat

**DOI:** 10.3390/plants15071095

**Published:** 2026-04-02

**Authors:** Xin Gao, Chunsheng Wang, Yumei Su, Hongzhi Zhang, Zhun Zhao, Lihong Wang, Zhong Wang, Junjie Han, Jianfeng Li, Yueqiang Zhang

**Affiliations:** 1 Institute of Crop Research, Academy of Agricultural Sciences of Xinjiang Uyghur Autonomous Region/Key Laboratory of Crop Biotechnology/Key Laboratory of Crop Ecophysiology and Farming System in Desert Oasis Region, Ministry of Agriculture and Rural Affairs, Urumqi 830091, China; gaoxin@xaas.ac.cn (X.G.); lihongwang@xaas.ac.cn (L.W.); wangzhongac@126.com (Z.W.); hanjunjie_xjnky@163.com (J.H.); 2 Xinjiang Engineering Research Center for Crop Chemical Regulation, Urumqi 830091, China; 3 College of Life Sciences, Xinjiang Agricultural University, Urumqi 830000, China

**Keywords:** spring wheat, low-nitrogen stress, MicroRNA, NUE, DELLA

## Abstract

Understanding the molecular mechanisms of low-nitrogen (LN) tolerance in common wheat (*Triticum aestivum* L.) is crucial for developing cultivars with improved nitrogen-use efficiency (NUE). In this study, a LN-tolerant cultivar (‘Xin Chun 29’, XC29) and a LN-sensitive cultivar (‘Xin Chun 11’, XC11) were used to investigate miRNA-mediated post-transcriptional regulation under LN stress. A total of 822 miRNAs were identified across root and grain tissues, including 104 known miRNAs and several tissue-specific candidates. In roots, *tae-miR395a* and *tae-miR444a* were significantly upregulated in XC29 under LN stress, putatively targeting an F-box ubiquitin ligase gene and glutathione reductase gene, respectively. In grains, the *tae-miR156/SBP* module was upregulated in XC29, whereas *tae-miR1118* and *tae-miR9778* were downregulated in XC11, potentially suppressing a receptor kinase gene and calmodulin gene. KEGG analysis revealed that target genes of differentially expressed miRNAs were significantly enriched in plant hormone signal transduction, ubiquitin-mediated proteolysis, and nitrogen metabolism. Notably, within the hormone signaling category, the gibberellin (GA) branch was highlighted by the co-targeting of DELLA genes by *tae-miR1130b-3p* and *tae-miR1120c-3p*. To elucidate this regulatory hub, a putative miRNA-target network centered on DELLA proteins was constructed, further underscoring the centrality of gibberellin signaling in the LN adaptation process. These findings suggest potential key miRNA-target modules contributing to LN adaptive responses and may provide useful genetic resources for molecular design breeding of nitrogen-efficient wheat.

## 1. Introduction

Common wheat (*Triticum aestivum* L.) is a cornerstone of global food security. In modern agricultural systems, achieving high yields is heavily dependent on nitrogen (N) fertilization. However, crop nitrogen use efficiency (NUE) remains notoriously low, with a substantial proportion of applied N (over 50%) lost to the environment, leading to economic waste and ecological damage such as eutrophication and greenhouse gas emissions [1,2]. The challenge of low nitrogen use efficiency (NUE) is particularly acute in arid and semi-arid regions. Taking Xinjiang, the largest provincial-level administrative region in China, as an example, its area accounts for approximately one-sixth of the nation’s total land area. However, nitrogen fertilizer utilization in wheat production in this region is concerning. Research indicates that under drip irrigation conditions, the optimal nitrogen application rate for spring wheat in Xinjiang is about 270 kg·hm^−2^, yet the average absorption and utilization rate of fertilizer nitrogen in the current season is only 19.79%. This figure is significantly lower than the national average, with a gap of about 30% [3,4]. The inefficient use of nitrogen fertilizer not only implies substantial resource waste and economic losses but also leads to severe environmental consequences, including nitrate leaching, ammonia volatilization, and soil acidification. Therefore, breeding wheat varieties with higher nitrogen use efficiency has become an urgent need to address these challenges and achieve sustainable agricultural development [5,6].

NUE is a complex quantitative trait governed by integrated physiological processes—uptake, translocation, assimilation, and remobilization—orchestrated by a multilayered molecular network [4]. Beyond the well-characterized roles of nitrate transporters and enzymes like glutamine synthetase [7], post-transcriptional regulation by microRNAs (miRNAs) has emerged as a critical mechanism for fine-tuning gene expression in response to N availability [8,9]. Key miRNA-N response modules are conserved across plants: *miR169* targets NF-YA transcription factors to modulate high-affinity nitrate transport [10]; *miR444a* positively regulates NRT expression and N accumulation [11,12]; and *miR167/ARF* and *miR393/AFB* modules integrate auxin signaling to shape root system architecture for optimal N foraging [13,14]. These findings establish miRNAs as pivotal molecular switches in plant N response.

A key downstream target of this miRNA-mediated regulation is hormone signaling, which translates nitrogen availability into adaptive growth responses [15,16]. Among the various hormone signaling pathways, gibberellin (GA) signaling plays a particularly important role in mediating growth responses to nitrogen availability [17,18]. GA promotes plant growth by targeting DELLA proteins for degradation via the ubiquitin-proteasome system [19]. And DELLA proteins act as growth repressors that integrate multiple environmental signals [20]. Nitrogen limitation typically suppresses GA signaling, leading to DELLA accumulation and reduced shoot growth—an adaptive strategy that reallocates resources to root development for enhanced nitrogen foraging [21]. However, the molecular mechanisms by which nitrogen status regulates GA signaling, particularly at the post-transcriptional level through miRNAs, remain poorly understood in wheat.

This knowledge gap is part of a broader under-exploration of miRNA-mediated nitrogen responses in wheat, especially in reproductive organs such as developing grains [22], and in locally adapted germplasm like Xinjiang spring wheat landraces [23]. Most studies have focused on root responses in seedlings, leaving the miRNA regulatory landscape in reproductive organs—particularly developing grains, the ultimate sink for N—largely unexplored [15]. Furthermore, while phytohormone signaling is implicated in N response, how miRNAs mediate crosstalk between N signaling and specific hormonal pathways, such as gibberellin (GA) signaling, to coordinate growth under stress is not well-defined [22].

To address these gaps, 29 Xinjiang spring wheat landraces were screened for their tolerance of LN through two-year field trials (2022–2023) under normal and low-nitrogen conditions. Based on grain yield, biomass, and physiological parameters, two cultivars with contrasting LN tolerance were identified: the tolerant cultivar ‘Xin Chun 29’ (XC29) and the sensitive cultivar ‘Xin Chun 11’ (XC11) (Supplementary Figure S1) [23]. Using this ideal pair, we combined high-throughput miRNA sequencing of root and grain tissues with bioinformatic and molecular analyses to: (i) systematically map the spatiotemporal miRNA expression profiles under LN stress; (ii) identify genotype- and tissue-specific miRNAs and predict their target genes, with a focus on hormone-related pathways; and (iii) construct core miRNA-target regulatory networks to elucidate the mechanistic basis of LN adaptation. This work provides a comprehensive resource and novel insights into the miRNA-mediated regulatory architecture underlying NUE in wheat, offering valuable targets for molecular breeding towards sustainable wheat production.

## 2. Results

### 2.1. Overview of miRNA Sequencing

To systematically identify miRNAs responsive to LN stress in Xinjiang spring wheat, small RNA deep sequencing was performed on 24 samples (two genotypes × two tissues × two treatments × three biological replicates), comprising two genotypes (LN-sensitive ‘XC11’ and LN-tolerant ‘XC29’) and two tissues (roots and grains) under normal nitrogen (N1) and LN treatments (sample parameters are provided in Table S1). The number of raw reads ranged from 11,748,032 to 34,014,504, with an average of approximately 17,321,590 raw reads per library. Sequencing yielded a total of 292.65 million clean reads, with each sample generating no fewer than 10.06 million clean reads (clean reads ranged from 10,055,819 to 15,928,767 after filtering) suitable for subsequent analyses (Table S2). The percentage of reads mapped to the Chinese Spring reference genome ranged from 13.21% to 79.30% (Table S3). These mapped reads comprised sequences annotated as rRNA, scRNA, snRNA, snoRNA, tRNA, Repbase, and unannotated sequences.

**Figure 1:**
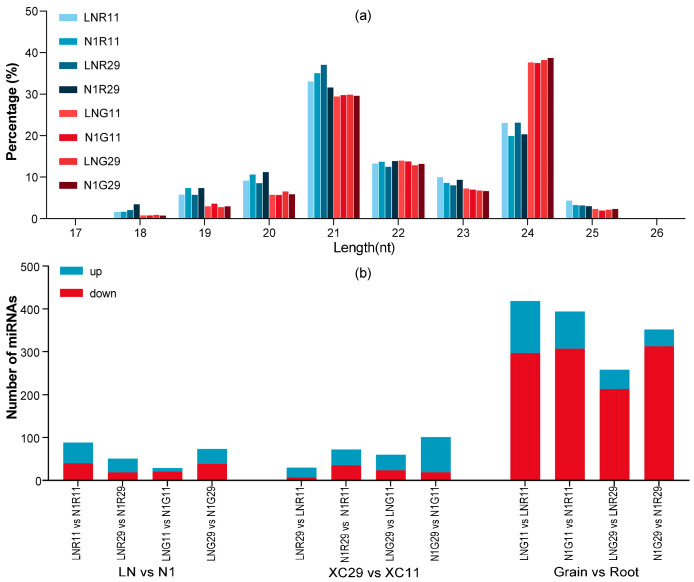
Small RNA Size Distribution and miRNA Expression Profiles Under Different Nitrogen Treatments, Genotypes, and Tissues. (**a**) Size distribution of small RNAs under different treatments and tissues. (**b**) Different nitrogen treatments and genotypes affect miRNA expression. Note: Plants were sampled under the following conditions: LN, low nitrogen stress; N1, normal nitrogen; R, root; G, grain; 11, nitrogen-sensitive cultivar Xin Chun 11; 29, low-nitrogen-tolerant cultivar Xin Chun 29. (**b**) *p* value < 0.05, |log2FC| ≥ 1.5.

To analyze the correlation between small RNA length and read abundance, we examined unique 13–31 nt reads. The length distribution of small RNAs showed prominent peaks at 21 nt and 24 nt across all samples. The 24 nt RNAs were the most abundant, accounting for 19.9% to 38.7% of total reads—a typical pattern for plant miRNAs [24]. Root samples were dominated by 21 nt small RNAs, while grain samples had the highest abundance of 24 nt ones. Under the same tissue and genotype conditions, LN and N1 treatments showed nearly identical length profile patterns, indicating short-term nitrogen stress had little effect on small RNA length distribution (Figure 1a). Moreover, no obvious cultivar-specific differences in small RNA size composition were observed between XC11 and XC29 under the same tissue and nitrogen treatment conditions.

### 2.2. Identification and Comparative Analysis of Known miRNAs

Through the identification and analysis of sequencing data from 24 samples, a total of 822 miRNAs were identified. Among these, 104 were known miRNAs, and 718 were newly predicted miRNAs (Table S5 for details). The known miRNAs could be classified into 74 distinct miRNA families. Abundance analysis of these families revealed that the *miR156* family exhibited the highest expression level, followed by the *miR9653*, *miR159*, *miR167*, *miR7757*, and *miR9672* families (Table S6 for details).

The expression levels varied considerably among different miRNA families. For instance, the total read count for the most abundant family, *miR156*, reached 79,176, whereas the read count for the least expressed family, *miR9781*, was only 1–2. Notably, even within the same family, distinct members displayed evident sequence variation and expression divergence. Taking the *miR9666* family as an example, the read count for member *miR9666c-5p* was merely 2, while that for another member, *miR9666a-3p*, was as high as 3199. Furthermore, the expression profiles of these family members varied across samples, suggesting that different members within the same miRNA family may perform specialized functions in response to tissue-specific expression or LN stress.

### 2.3. Analysis of Tissue-Specific and Differentially Expressed miRNAs (DEMs)

Across comparisons of tissue (grain vs. root), genotype (‘XC29’ vs. ‘XC11’), and nitrogen level (LN vs. N1), numerous differentially expressed miRNAs (DEMs) were identified (Figure 1b). Within the same genotype and tissue, nitrogen-level comparisons (A_vs._B, C_vs._D, E_vs._F, G_vs._H) yielded 29–88 DEMs, with more miRNAs upregulated than downregulated in roots, whereas the opposite trend was observed in grains [24]. This result aligns with the broader observation that miRNA expression tends to be more active and predominantly upregulated in roots, whereas downregulation prevails in grains. Genotype comparisons under the same tissue and nitrogen condition (C_vs._A, D_vs._B, G_vs._E, H_vs._F) identified 30–101 DEMs—slightly higher than nitrogen-treatment comparisons. Notably, in grains, the low-nitrogen-tolerant cultivar ‘XC29’ showed a greater number of upregulated miRNAs, which may contribute to its nitrogen assimilation efficiency for more stable yield under LN stress. By integrating previously reported low-nitrogen-responsive miRNAs (Tables S7–S9), we defined a set of core DEMs: eight in roots and six in grains, which include *tae-miR395a* and *tae-miR9653a-3p*. Expression trends for the selected candidates were further validated by RT-qPCR (Section 2.5).

**Figure 2:**
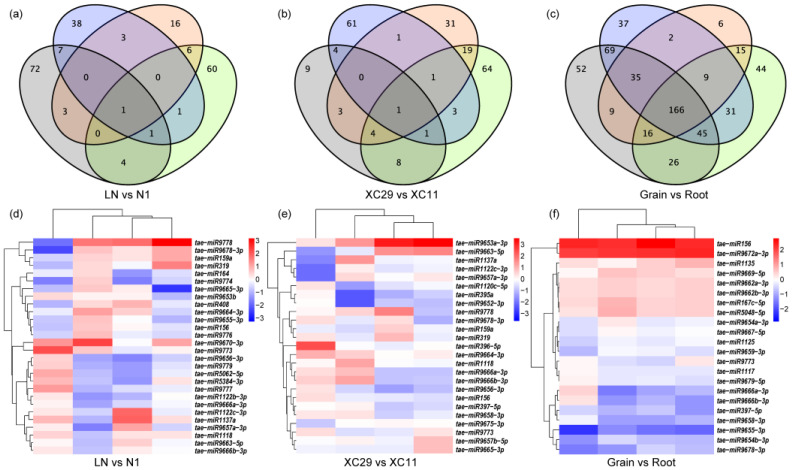
Venn diagram and expression levels of differentially expressed miRNAs (DEMs) between genotypes and organs. (**a**–**c**) Venn diagram of differentially expressed miRNAs (DEMs) between genotypes and organs. (**d**–**f**) Expression Levels of Commonly Differentially Expressed miRNAs (DEMs). Note: (**d**–**f**) blue indicating downregulation, red indicating upregulation.

Cross-analysis of miRNA expression profiles (Figure 2a–c, Tables S7–S9) revealed that only one known DEM (*tae-miR9778*) was shared among the four nitrogen treatment comparison groups, and only one novel DEM (*novel_miR_524*) was shared among the four genotype comparison groups. By contrast, inter-tissue comparisons (Grain vs. Root) shared 166 DEMs, suggesting that nitrogen- and genotype-driven miRNA regulation is highly specific and the existence of numerous conserved regulatory miRNAs between tissues whose expression is relatively unaffected by nitrogen treatment and genetic background. Clustering analysis revealed that root samples (A–D) and grain samples (E–H) formed separate clusters. Nitrogen treatment and genotype further contributed to distinct subclustering patterns within each tissue (Figure 2d–f, Table S10).

### 2.4. Target Gene Prediction and Enrichment Analysis of DEMs

**Figure 3:**
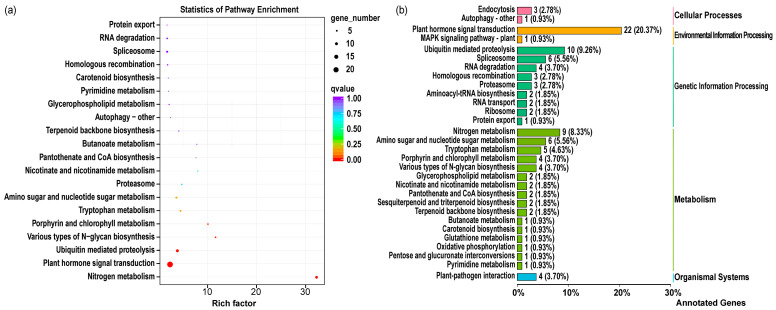
KEGG analysis of target genes of differentially expressed miRNAs (DEMs). (**a**) KEGG enrichment of target genes of differentially expressed miRNAs (DEMs). (**b**) KEGG class of target genes of differentially expressed miRNAs (DEMs). Note: (**a**) Dot color and size indicate enrichment significance (*p* < 0.05) and gene number, respectively. (**b**) Pathways are grouped by functional categories.

To explore the regulatory mechanism of differentially expressed miRNAs (DEMs) during wheat adaptation to low-nitrogen (LN) stress, KEGG enrichment analysis revealed that the DEMs were significantly enriched in key pathways such as plant hormone signal transduction and ubiquitin-mediated proteolysis (Figure 3a). Within the plant hormone signal transduction pathway (Figure 3b), eight known miRNAs (e.g., tae-miR1137a, tae-miR171b) were identified to target members of the DELLA protein family (e.g., GAI-like, SLR1, RGL2), while tae-miR156 and tae-miR5048-5p targeted protein phosphatase 2C 53 and the gibberellin receptor TaGID1, respectively. Concurrently, in the ubiquitin-mediated proteolysis pathway, four other known miRNAs (e.g., tae-miR9657b-5p, tae-miR1121) were found to target degradation system components such as E2/E3 ubiquitin ligases and F-box proteins. Taken together, these results indicate that during wheat’s response to LN stress, the gibberellin signaling pathway is primarily regulated through the synergistic action of specific known miRNAs within the plant hormone signal transduction and ubiquitin-mediated proteolysis pathways. This coordinated regulation modulates DELLA protein stability and gibberellin perception, thereby maintaining gibberellin signaling homeostasis and providing crucial regulatory support for wheat to adapt to LN stress.

**Figure 4:**
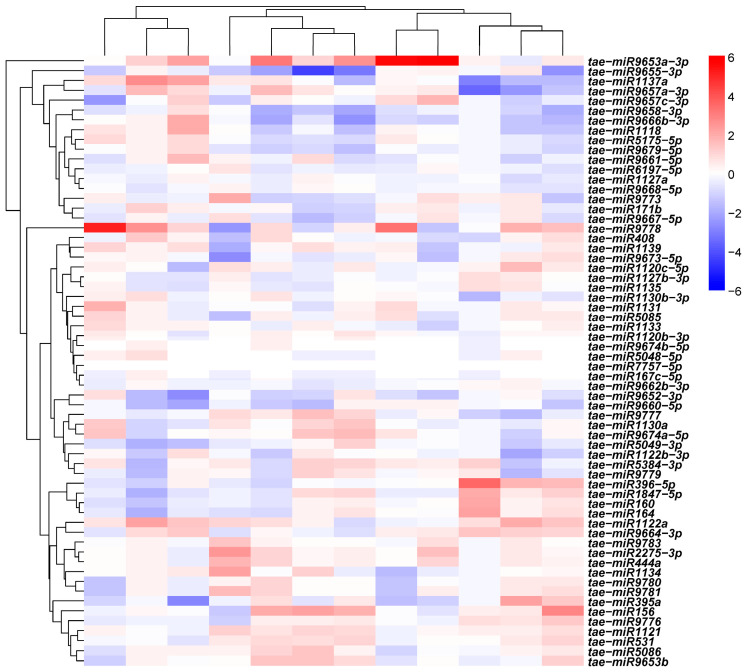
Clustering analysis of differentially expressed miRNAs (DEMs) related to nitrogen metabolism.

To gain deeper mechanistic insights, we narrowed our focus to 27 nitrogen metabolism-related DEMs exhibiting robust expression levels (TPM > 20). The expression heatmap revealed complex and highly context-specific patterns across different nitrogen treatments, tissue types, and genotypic backgrounds (Figure 4). Notably, *tae-miR156* exhibited the most pronounced upregulation under LN stress (log_2_FC = 3.92), whereas *tae-miR1118* showed strong downregulation specifically in grains (log_2_FC = –4.42). Based on target prediction results for these DEMs, we constructed an integrated miRNA-target gene regulatory network under LN stress (Supplementary Figure S2). Network analysis identified several functionally distinct regulatory modules:

Hormonal signal integration module: Eleven miRNAs, including *tae-miR1133* and *tae-miR171b*, were predicted to co-target DELLA proteins (K14494), which are core repressors of gibberellin (GA) signaling. Notably, two specific miRNAs—*tae-miR1130b-3p* and *tae-miR1120c-5p*—showed particularly strong differential expression under LN stress and were predicted to target multiple DELLA family members (*TaDELLA1*, *TaDELLA2*). The upregulation of these miRNAs in the LN-tolerant cultivar XC29 suggests that enhanced repression of DELLA genes may alleviate GA signaling suppression, enabling better growth maintenance under nitrogen limitation. Thus, this convergence may represent an important node orchestrating crosstalk between GA, other phytohormones, and nitrogen signaling pathways. Auxin response module: *tae-miR1847-5p* and *tae-miR395a* jointly targeted GH3, a key auxin-responsive promoter gene implicated in hormonal homeostasis. Stress signaling and protein modification module: Multiple miRNAs targeted genes encoding kinases [25], DnaJ molecular chaperones, F-box proteins (involved in ubiquitination), and ABA signaling components. These targets collectively span stress signal transduction, protein folding, and proteolytic degradation pathways [23,26]. The identification of DELLA proteins as a hub in the miRNA-target network is particularly significant, as DELLA proteins are known to integrate multiple hormone signaling pathways, including GA, auxin, and abscisic acid [27]. By targeting DELLA genes, these miRNAs may serve as master regulators that coordinate hormone crosstalk in response to nitrogen stress. Therefore, this miRNA-mediated regulation of GA signaling represents a previously underappreciated layer of control in wheat nitrogen response.

In summary, integrated target prediction and enrichment analyses established that plant hormone signaling, ubiquitination-mediated proteolysis, and nitrogen metabolism constitute the mechanistic core pathways mediating miRNA-regulated responses to LN stress. Furthermore, the identification of hub genes such as *DELLA* through regulatory network construction provides clear mechanistic insights and a robust foundation for understanding the molecular circuitry underlying plant adaptation to nitrogen deficiency.

### 2.5. Confirmation of miRNAs by RT-qPCR

**Figure 5:**
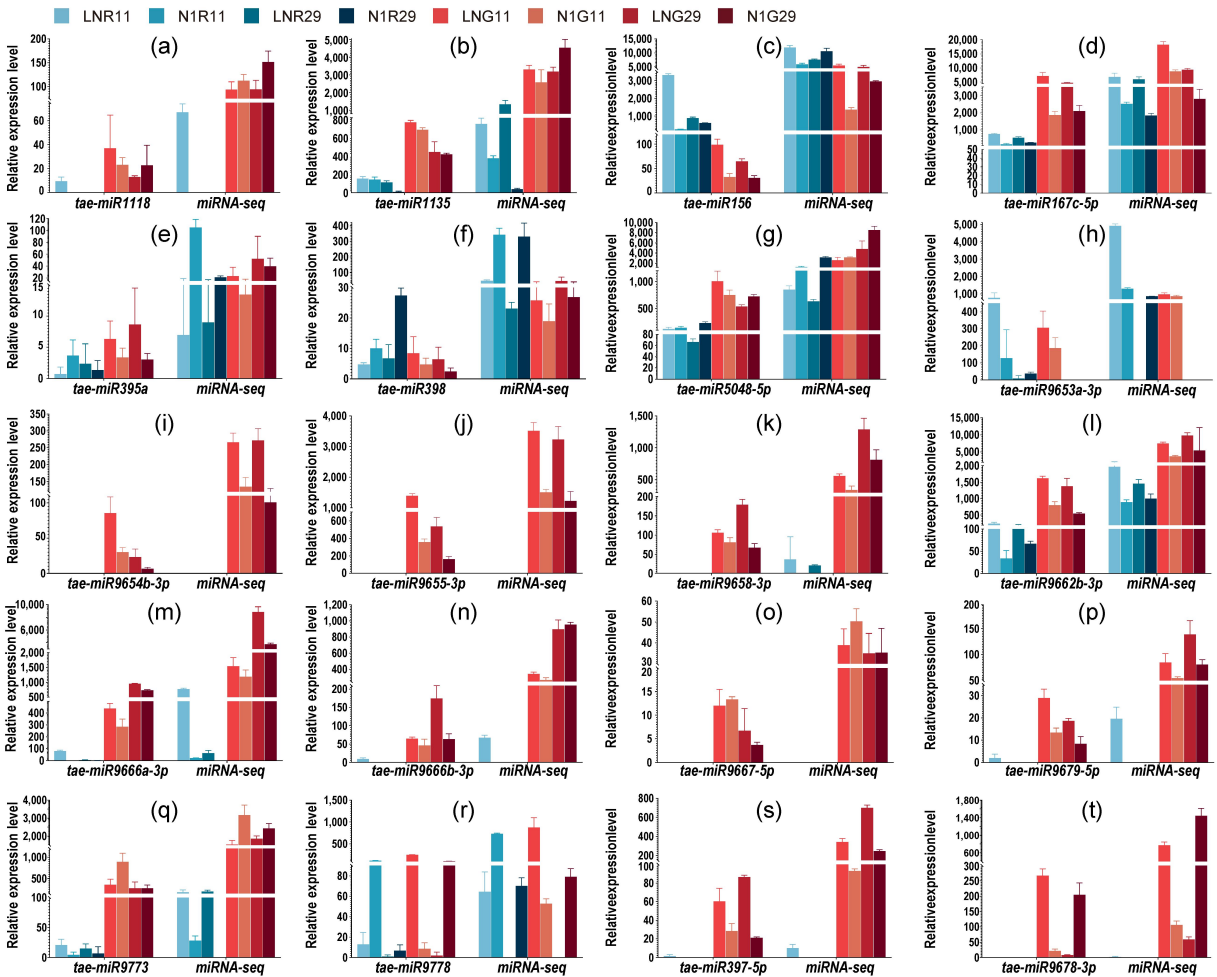
RT-qPCR validation of select miRNAs Expression. (**a**–**t**) Represent the miRNAs miR1118, miR1135, miR156, miR167c-5p, miR395a, miR398, miR5048-5p, miR9653a-3p, miR9654b-3p, miR9655-3p, miR9658-3p, miR9662b-3p, miR9666a-3p, miR9666b-3p, miR9667-5p, miR9679-5p, miR9673, miR9778, miR397-5p, and miR9678-3p.

To validate the reliability of high-throughput sequencing results, we quantified the expression of 20 candidate miRNAs using stem-loop RT-qPCR (Figure 5; primers are listed in Table S14). Among these candidates, 14 were core DEMs (eight in roots and six in grains). These candidates were selected to represent diverse expression patterns, including nitrogen-responsive DEMs (both up- and down-regulated), tissue-specific miRNAs, and genotype-dependent variants. The RT-qPCR results were strongly concordant with the miRNA-seq data (Pearson’s r = 0.89, *p* < 0.001), and the expression by sequencing and RT-qPCR performed consistent upregulate and downregulate patterns. These results confirmed the accuracy, reproducibility, and biological relevance of the sequencing data, providing a robust foundation for subsequent functional studies, including miRNA overexpression and target validation experiments to dissect their mechanistic roles in wheat adaptation to LN stress.

## 3. Discussion

Plants have evolved considerable phenotypic plasticity to adapt to environmental variability. Our miRNA profiling under LN stress suggests that miRNAs act as precise post-transcriptional switches in this adaptive process in wheat [28]. We identified 822 miRNAs, of which 27 were significantly associated with nitrogen metabolism, displaying clear tissue specificity and genotype dependence. In roots, *tae-miR395a* and *tae-miR444a* were markedly upregulated, targeting *TraesCS7D03G0946700* (F-box protein gene) and *TaGRV2* (glutathione reductase gene), implying roles in protein degradation and redox homeostasis. In grains, by contrast, *tae-miR1118* and *tae-miR9778* were downregulated, potentially suppressing a receptor kinase gene and *TaCaM2-1* (calmodulin gene) to affect nitrogen assimilation enzyme activity. These distinct patterns reflect organ-specific strategies: roots prioritize nutrient uptake and stress defense, whereas grains focus on the synthesis of storage compounds and efficient nitrogen remobilization [25,29,30,31]. Notably, the LN-tolerant cultivar Xin Chun 29 showed more upregulated miRNAs in grains (e.g., *tae-miR9653a-3p*), suggesting that genotypic differences arise from the resilience of entire regulatory networks rather than individual miRNAs [32,33,34]. Target genes were significantly enriched in three interconnected KEGG pathways: “*Plant hormone signal transduction*” (KO04075), “*Ubiquitin-mediated proteolysis*” (KO04120), and “*Nitrogen metabolism*” (KO00910), aligning with conserved regulatory roles across rice, maize, and soybean [35,36,37]. This multi-layer architecture, coordinated through miRNA nodes, forms a sophisticated post-transcriptional network underpinning wheat adaptation to LN stress [38].

Analysis of core DEMs underscores the central role of hormone signaling as a hub that integrates nitrogen signals with growth responses. Multiple DEMs (*tae-miR1130b-3p* and *tae-miR1120c-5p*) collectively target DELLA proteins (K14494), core repressors of gibberellin (GA) signaling, paralleling the miRNA-*DELLA* module reported in *Phyllostachys edulis* [39,40]. Our data show these miRNAs undergo marked expression changes under LN, suggesting nitrogen stress reprograms GA signaling to influence elongation growth and resource allocation. This provides a molecular perspective on the dwarfing phenotypes observed in “fertilizer-saving” varieties [6]. Furthermore, *tae-miR9653a-3p*, specifically upregulated in the LN-tolerant cultivar XC29, targets auxin response factor *ARF8* (*TraesCS3B03G046100*), aligning with the conserved *miR167/ARF8* module regulating lateral root development in response to nitrogen availability [13,27]. Beyond GA and auxin, our target set was enriched in abscisic acid (ABA) and jasmonic acid (JA) pathways, including the predicted target *SRC2*, indicating wheat employs a coordinated multi-hormone defense network [41]. We also identified wheat-specific relationships, such as *tae-miR9778* targeting *TaCaM2-1*, a calmodulin gene rarely documented in nitrogen responses of other crops. By mapping these interactions (Supplementary Figure S2), our work supports the existence of conserved modules and suggests wheat-specific networks that orchestrate nitrogen-stress adaptation, providing candidate targets for improving nitrogen-use efficiency.

One of the central findings of this study is the identification of a miRNA-DELLA regulatory module that links nitrogen signaling to GA pathway modulation. DELLA proteins are core repressors of GA signaling that integrate environmental cues to regulate growth [42]. Multiple miRNAs, particularly *tae-miR1130b-3p* and *tae-miR1120c-5p*, were predicted to co-target DELLA genes, and their upregulation in the LN-tolerant cultivar XC29 suggests that enhanced DELLA repression may partially relieve GA suppression, enabling better growth maintenance under nitrogen limitation. This miRNA-mediated fine-tuning of GA signaling represents a mechanistic link between nitrogen perception and growth regulation not previously described in wheat. Similar miRNA-DELLA modules have been reported in other species [43], suggesting evolutionary conservation, though the specific miRNAs involved appear genotype-specific. Future experimental validation of these interactions will be essential to confirm their functional roles.

A key finding is the marked divergence in miRNA expression and LN response patterns between roots and grains, suggesting that these tissues play specialized roles in whole-plant nitrogen management. In grains, activation of the *tae-miR156/SBP* module may promote nitrogen remobilization during grain filling by modulating carbon-nitrogen balance genes, aligning with reports on glutamate synthase expression changes under nitrogen stress [43,44]. In roots, strong upregulation of *tae-miR444a*, previously shown in rice to enhance nitrate transcription expression, suggests a conserved role in improving nitrogen acquisition [12]. Notably, root-enriched miRNAs (*miR444a*, *miR395a*) and grain-enriched miRNAs (*miR156*, *miR1118*) exhibit clear functional differentiation: root miRNAs bias toward nutrient uptake and primary assimilation, whereas grain miRNAs associate with storage-compound synthesis and redistribution, providing precise spatiotemporal control over nitrogen flow. Such differentiation may link to long-distance signaling, wherein shoots modulate root responses through mobile miRNAs or hormonal signals [45,46]. Future studies should explore how these tissue-specific miRNAs interact with systemic networks to optimize nitrogen allocation under combined stresses in complex field environments [47]. Furthermore, our data reveal a potential miRNA-mediated mechanism for adapting to LN stress through modulation of the GA signaling pathway. The observed down-regulation of miRNAs targeting DELLA proteins, coupled with the up-regulation of miRNAs targeting the GA receptor GID1, suggests a strategic shift in the plant’s growth-defense balance. Under LN stress, the plant appears to suppress GA sensitivity (via GID1 targeting) and enhance DELLA activity (via reduced miRNA-mediated cleavage), leading to growth inhibition. This molecular reprogramming is consistent with the known role of DELLA proteins in promoting stress tolerance [48]. Additionally, as this analysis was performed at a single time point (15 days after anthesis), incorporating multiple developmental stages and experimental validation of predicted miRNA-target interactions will be essential to fully capture dynamic regulatory trajectories and translate promising candidates into molecular design breeding [49,50].

The gibberellin (GA) and DELLA signaling pathways are the core regulatory pathways for wheat adaptation to low-nitrogen stress, and miRNA-mediated post-transcriptional regulation is a key link in maintaining the homeostasis of this pathway. As confirmed in the Section 2.4, known miRNAs (such as tae-miR1137a and tae-miR9657b-5p) participate in pathway regulation by directly targeting DELLA proteins and regulating their degradation, respectively. The key novel miRNAs identified in this study further improve this regulatory network, among which novel_miR_146 and novel_miR_625 play particularly prominent roles. Novel_miR_146 synergistically targets DELLA protein GAI-like with the known miRNA tae-miR1137a, which is similar to the regulatory mode of miR159 targeting DELLA proteins in Arabidopsis thaliana reported in previous studies [51]. However, its specific expression under wheat low-nitrogen stress suggests that it may be a wheat-specific GA pathway regulatory factor. Novel_miR_625 targets E3 ubiquitin-protein ligase SINA-like 7, and synergistically regulates DELLA protein stability with the known miRNA tae-miR9657b-5p (targeting ubiquitin-conjugating enzyme E2 23) from different links of ubiquitination degradation [52]. This is different from the mechanism by which miR396 regulates DELLA degradation in rice, reflecting the species specificity of GA pathway regulation in gramineous crops. In summary, key novel miRNAs and known miRNAs form a synergistic network of “direct targeting + degradation regulation”, providing support for the precise regulation of the GA pathway under wheat low-nitrogen stress and enriching the miRNA regulatory theory of the GA signaling pathway in gramineous crops.

## 4. Materials and Methods

### 4.1. Plant Materials

Spring wheat cultivars Xin Chun 11 (XC11; nitrogen-sensitive) and Xin Chun 29 (XC29; low-nitrogen-tolerant) (Supplementary Figure S1) [23], two elite cultivars cultivated in Xinjiang, were used in this study. Seeds were provided by the Crop Research Institute, Xinjiang Academy of Agricultural Sciences.

### 4.2. Measurements and Methods

#### 4.2.1. Plant Growth Conditions and Experimental Design

The experiment was conducted using a pot culture system. Growth chamber conditions were maintained as follows: light intensity of 300–320 µmol/m^2^/s, relative air humidity of 65%, and day/night cycles of 14 h/24 °C and 10 h/18 °C, respectively. Uniform and plump seeds of XC11 and XC29 were surface-sterilized by stirring in 5% sodium hypochlorite for 10 min, rinsed 3–4 times with distilled water, and then placed on filter paper in Petri dishes for germination. When roots reached 1–2 cm in length, seedlings were transferred to plastic pots filled with peat substrate. The main physicochemical properties of the peat substrate were as follows: pH ≈ 7.1, organic matter 16.4 g/kg, total N 0.9 g/kg, available N 63.5 mg/kg, and inorganic N accumulation 112.5 kg/hm^2^. Two nitrogen treatments were established: low nitrogen stress (LN; 0 mmol/L nitrogen source throughout the growth period) and normal nitrogen (N1; 3 mmol/L nitrogen source supplied four times during the growth period), with three biological replicates per treatment and 10 pots per replicate. NH_4_NO_3_ was used as the nitrogen source for N1 treatment. Samples of wheat grains (G) and roots (R) were collected at 15 days after anthesis (n = 15 plants per group), immediately frozen in liquid nitrogen, and stored at –80 °C until use.

Subsequent comparative analyses were performed among three groups: LN vs. N1; G vs. R; and XC29 vs. XC11. RNA samples were analyzed as three independent biological replicates rather than pooled samples.

For simplicity, the following abbreviations are used throughout this study: LN, low-nitrogen stress; N1, normal nitrogen; R, root; G, grain; 11, nitrogen-sensitive cultivar Xin Chun 11; 29, low-nitrogen-tolerant cultivar Xin Chun 29.

#### 4.2.2. RNA Extraction and Small RNA Library Construction

Total RNA was extracted using the RNA Mini Kit, and genomic DNA was removed using DNase I (Thermo Fisher Scientific, Waltham, MA, USA), following the manufacturer’s protocols. RNA yield and integrity were assessed by 1.0% agarose gel electrophoresis, NanoDrop spectrophotometry (Thermo Fisher Scientific, Waltham, USA), and Agilent 2100 Bioanalyzer analysis (Agilent Technologies, Santa Clara, CA, USA). Equal quantities (5 µg) of total RNA from three biological replicates of each treatment were pooled for small RNA library construction. Sequencing was performed on the Illumina NovaSeq 6000 platform (Illumina, San Diego, CA, USA) using a single-end 50 Bp (SE50) model (Biomarker Technologies, Beijing, China).

#### 4.2.3. Bioinformatics Analysis

Raw sequencing reads were first subjected to quality assessment using FastQC (v0.11.9) (https://www.bioinformatics.babraham.ac.uk/projects/fastqc/Official (accessed on 29 October 2025)). Subsequently, adapter sequences were trimmed, and reads shorter than 18 nt or longer than 30 nt, low-quality reads (with >50% of bases having Phred scores <20), and reads containing ambiguous ‘N’ bases were removed to generate high-quality clean reads. High-quality clean reads were mapped to the wheat reference genome (IWGSC RefSeq v2.1). Known miRNAs were annotated using miRBase v22 (https://www.mirbase.org/). Novel miRNAs were predicted using miRDeep2 with folding energy ≤–20 kcal/mol (https://deepwiki.com/rajewsky-lab/mirdeep2 (accessed on 29 October 2025)). Raw read counts for each miRNA were calculated using miRDeep2. To eliminate biases arising from sequencing depth differences, normalization was performed using transcripts per million (TPM). Only miRNAs with mean TPM > 1 in at least one group were retained for subsequent analyses to reduce noise from low-abundance transcripts. Differential expression analysis was conducted on the BMKCloud platform (https://www.biocloud.net/) using DESeq2 [53]. The Benjamini–Hochberg method was applied for multiple testing correction. Differentially expressed miRNAs (DEMs) were identified with a false discovery rate (FDR) < 0.01, and their expression patterns were analyzed [21]. Comparisons were performed among the following groups: LN versus N1, grain versus root, and XC29 versus XC11, to screen DEMs and analyze their expression patterns, regulatory relationships, and fold changes. For target gene prediction, psRNATarget (https://www.zhaolab.org/psRNATarget/ (accessed on 29 October 2025)) was employed with the following parameters: expectation value ≤ 3.0 and maximum unpaired energy (UPE) ≤ 25. Functional annotation was performed using Gene Ontology (GO) and Kyoto Encyclopedia of Genes and Genomes (KEGG) enrichment analyses (using clusterProfiler v4.6).

#### 4.2.4. RT-qPCR Confirmation

Candidate miRNAs for validation were selected based on their expression patterns: upregulated candidates included *miR1133, miR1136, miR167a, miR2275, miR444a, miR156, miR399,* and *miR408*; downregulated candidates included *miR1118, miR160, miR164,* and *miR319*. Primers were designed using miRPrimer ver.2.0 (15 January 2019, Center for Bioinformatics, Peking University, Beijing, China; https://sourceforge.net/projects/miRprimer/ (accessed on 29 October 2025)); specific primer sequences are listed in Supplementary Table S2. geNorm and NormFinder validation of reference gene stability.

geNorm Results: The M value for U6 was calculated to be 0.21, which is significantly lower than the accepted threshold of 1.5. This indicates that U6 expression was highly stable across both the control and LN treatment groups.

NormFinder Results: The stability value for U6 was 0.14, ranking it as the most stable gene among the tested candidates under our experimental conditions.

U6 small nuclear RNA (snRNA) served as the internal reference gene. Reverse transcription of miRNAs was performed using the TaqMan MicroRNA Reverse Transcription Kit (ver.4.0, 20 March 2022, Thermo Fisher Scientific, Waltham, MA, USA; https://www.thermofisher.com/ (accessed on 29 October 2025)). Real-time quantitative PCR (qRT-PCR) was conducted using SYBR Green Master Mix (ver.2.0, 30 June 2021, Roche Diagnostics GmbH, Mannheim, Germany; https://www.roche.com/) on an ABI 7500 Real-Time PCR System (ver.2.3, 10 September 2020, Applied Biosystems, Foster City, CA, USA; https://www.thermofisher.com/us/en/home/brands/applied-biosystems.html (accessed on 29 October 2025)) under the following conditions: 95 °C for 30 s, followed by 40 cycles of 95 °C for 5 s and 60 °C for 34 s. Three biological replicates were included for each sample. Relative expression levels were calculated using the 2^^(–ΔΔCt)^ method (also known as the comparative Ct method, ver.1.0, 2001, Kenneth J. Livak and Thomas D. Schmittgen; https://www.ncbi.nlm.nih.gov/pubmed/11846609 (accessed on 29 October 2025) [54], which is a standard method for relative quantification of gene expression in qRT-PCR experiments.

### 4.3. Data Analysis

The test data were used for variance analysis (ANOVA), employing the least significant difference (LSD) method for multiple comparisons to assess the significance of differences. The level of *p* < 0.05 was considered statistically significant, while *p* < 0.01 denoted a highly significant distinction. Statistical visualizations were performed using R ver.4.3.1 (15 March 2023, R Foundation for Statistical Computing, Vienna, Austria; https://www.r-project.org/). Heatmaps were generated using the ggplot2 package (ver.3.4.4, 12 June 2023, Hadley Wickham, Winston Chang, Lionel Henry, Thomas Lin Pedersen, Kohske Takahashi, Claus Wilke, Kara Woo, Hiroaki Yutani, Dewey Dunnington; https://ggplot2.tidyverse.org/) [55]. Data consistency among biological replicates was assessed using Pearson correlation coefficients (|r| > 0.85 was considered acceptable), which was implemented using the cor.test function in R ver.4.3.1 (15 March 2023, R Foundation for Statistical Computing, Vienna, Austria; https://www.r-project.org/). Figure generation was conducted using GraphPad Prism ver.9.0 (October 2020, Dotmatics Corporation, Boston, MA, USA), and regulatory networks of core differentially expressed miRNAs (DEMs) and their target genes were constructed using Cytoscape ver.3.9.1 (1 July 2021 Cytoscape Consortium, Institute for Systems Biology, Seattle, WA, USA; https://cytoscape.org/).

## 5. Conclusions

This study provides a comprehensive miRNA expression atlas in root and grain tissues of Xinjiang spring wheat under low-nitrogen stress, revealing complex, tissue-specific, and genotype-dependent regulatory responses. We identified key miRNA-target modules linked to nitrogen metabolism, hormone signaling, and protein degradation, with particular emphasis on a miRNA-DELLA axis that may fine-tune gibberellin signaling to balance growth under nitrogen limitation. The convergence of multiple miRNAs on DELLA genes highlights this node as a potential master regulator of nitrogen-stress adaptation. These findings contribute to our understanding of nitrogen-use efficiency in wheat and provide promising candidate targets for breeding nitrogen-efficient cultivars tailored to arid environments.

## Data Availability

The data that support the findings of this study are available upon request from the corresponding author. All raw sequencing data generated in this study have been uploaded to the National Genomics Data Center (NGDC, https://ngdc.cncb.ac.cn/) under the accession number PRJCA059947. The data will be publicly available after the manuscript is published.

## Supplementary Materials

The following supporting information can be downloaded at: https://www.mdpi.com/article/10.3390/plants15071095/s1.

## References

[1] Mălinaş A., Vidican R., Rotar I., Mălinaş C., Moldovan C.M., Proorocu M. (2022). Current Status and Future Prospective for Nitrogen Use Efficiency in Wheat (*Triticum Aestivum* L.). Plants.

[2] Zhang S., Ji Z., Jiao W., Shen C., Qin Y., Huang Y., Huang M., Kang S., Liu X., Li S. (2025). Natural Variation of OsWRKY23 Drives Difference in Nitrate Use Efficiency between Indica and Japonica Rice. Nat. Commun..

[3] Yin H., Kong L., Wang R., Che Z., Liu J., Jiang G., Xie B., Jiang F., Zhang T. (2023). Fate and Efficiency of Fertilizer Nitrogen in Spring Wheat Production under Drip Irrigation Based on the 15N Tracing Method. J. Plant Nutr. Fertil..

[4] Chattha M.S., Ali Q., Haroon M., Afzal M.J., Javed T., Hussain S., Mahmood T., Solanki M.K., Umar A., Abbas W. (2022). Enhancement of nitrogen use efficiency through agronomic and molecular based approaches in cotton. Front. Plant Sci..

[5] Guo M., Wang Q., Zong Y., Nian J., Li H., Li J., Wang T., Gao C., Zuo J. (2021). Genetic Manipulations of TaARE1 Boost Nitrogen Utilization and Grain Yield in Wheat. J. Genet. Genom..

[6] Das S., Sathee L. (2023). miRNA Mediated Regulation of Nitrogen Response and Nitrogen Use Efficiency of Plants: The Case of Wheat. Physiol. Mol. Biol. Plants.

[7] Gayatri, Jayaraman K., Sinha S.K., Roy P., Mandal P.K. (2021). Comparative Analysis of GS2 and Fd-GOGAT Genes in Cultivated Wheat and Their Progenitors Under N Stress. Plant Mol. Biol. Rep..

[8] Qiang B., Yan Z., Zhang X., Cheng M., Wu Y., Tang N., Timbang B.C., Cai T., Liu E., Zhao X. (2025). Elevating Crop Productivity and Sustainability: The Role of Organic Fertilizers in Shaping Wheat’s Nitrogen Economy and Photosynthetic Response. Eur. J. Agron..

[9] Xu W., Chen Y., Niu Y., Liu B., Du D., Ning X., Huan T., Zhou Y., Ke W., Miao L. (2025). An Incoherent Feed-Forward Loop Coordinates Nitrate Uptake and Tillering in Wheat. Mol. Plant.

[10] Chakraborty A., Sharma S., Pandey G.K., Bhatia S., Prasad M. (2025). Delineating microRNA169-*Nuclear Factor Y-Subunit A* Module for Its Potential Implications in Crop Improvement. Plant Cell Environ..

[11] Hou M., Yu M., Li Z., Ai Z., Chen J. (2021). Molecular Regulatory Networks for Improving Nitrogen Use Efficiency in Rice. Int. J. Mol. Sci..

[12] Yan Y., Wang H., Hamera S., Chen X., Fang R. (2014). miR444a Has Multiple Functions in the Rice Nitrate-signaling Pathway. Plant J..

[13] Wang Y., Li K., Chen L., Zou Y., Liu H., Tian Y., Li D., Wang R., Zhao F., Ferguson B.J. (2015). MicroRNA167-Directed Regulation of the Auxin Response Factors *GmARF8a* and *GmARF8b* Is Required for Soybean Nodulation and Lateral Root Development. Plant Physiol..

[14] Vidal E.A., Araus V., Lu C., Parry G., Green P.J., Coruzzi G.M., Gutiérrez R.A. (2010). Nitrate-Responsive miR393/*AFB3* Regulatory Module Controls Root System Architecture in *Arabidopsis thaliana*. Proc. Natl. Acad. Sci. USA.

[15] Nagel R. (2020). Gibberellin Signaling in Plants: Entry of a New MicroRNA Player. Plant Physiol..

[16] Gazara R.K., Moharana K.C., Bellieny-Rabelo D., Venancio T.M. (2018). Expansion and Diversification of the Gibberellin Receptor GIBBERELLIN INSENSITIVE DWARF1 (GID1) Family in Land Plants. Plant Mol. Biol..

[17] Blanco-Touriñan N., Serrano-Mislata A., Alabadí D. (2020). Regulation of DELLA Proteins by Post-Translational Modifications. Plant Cell Physiol..

[18] Mu X., Chen Q., Wu X., Chen F., Yuan L., Mi G. (2018). Gibberellins Synthesis Is Involved in the Reduction of Cell Flux and Elemental Growth Rate in Maize Leaf under Low Nitrogen Supply. Environ. Exp. Bot..

[19] Wu K., Xu H., Gao X., Fu X. (2021). New Insights into Gibberellin Signaling in Regulating Plant Growth–Metabolic Coordination. Curr. Opin. Plant Biol..

[20] Kong L., Zhang Y., Du W., Xia H., Fan S., Zhang B. (2021). Signaling Responses to N Starvation: Focusing on Wheat and Filling the Putative Gaps With Findings Obtained in Other Plants. A Review. Front. Plant Sci..

[21] Waheed A., Iqbal M.S., Sarfraz Z., Hou J., Wei Y., Song B., Cheng S. (2025). Watkins Wheat Landraces Decode Nitrogen-Driven Biomass Trade-Offs: GWAS Exposes Root-Shoot Dialectics and Elite Landraces for Resilient Agriculture. Front. Plant Sci..

[22] Luo P., Di D., Wu L., Yang J., Lu Y., Shi W. (2022). MicroRNAs Are Involved in Regulating Plant Development and Stress Response through Fine-Tuning of TIR1/AFB-Dependent Auxin Signaling. Int. J. Mol. Sci..

[23] Gao X., Wang Y., Zhu T., Li J., Wang Z., Shi J., Wang C., Zhang H., Wang L., Fan Z. (2024). Influence of different nitrogen application levels on the grain filling rate and yields of spring wheat. Xinjiang Agric. Sci..

[24] Agarwal S., Mangrauthia S.K., Sarla N. (2015). Expression Profiling of Iron Deficiency Responsive microRNAs and Gene Targets in Rice Seedlings of Madhukar x Swarna Recombinant Inbred Lines with Contrasting Levels of Iron in Seeds. Plant Soil.

[25] Zhang Y., Ma C., Li X., Hou X., Wang Z., Zhang J., Zhang C., Shi X., Duan W., Guo C. (2025). Wheat Tae-MIR1118 Constitutes a Functional Module With Calmodulin TaCaM2-1 and MYB Member TaMYB44 to Modulate Plant Low-N Stress Response. Plant Cell Environ..

[26] Vieira J.G.P., Duarte G.T., Barrera-Rojas C.H., Matiolli C.C., Viana A.J.C., Canesin L.E.D., Vicentini R., Nogueira F.T.S., Vincentz M. (2023). Regulation of *PYR/PYL/RCAR* ABA Receptors mRNA Stability: Involvement of miR5628 in Decay of *PYL6* mRNA. bioRxiv.

[27] Trösch R. (2024). A microRNA Switch for Nitrogen Deficiency. Nat. Plants.

[28] Sharma S., Singh D., Joon R., Shukla V., Singh A.P., Singh P., Mantri S., Pandey A.K. (2024). System Analysis of Differentially Expressed miRNAs in Hexaploid Wheat Display Tissue-Specific Regulatory Role During Fe-Deficiency Response. Plant Mol. Biol. Rep..

[29] Jagadhesan B., Das S., Singh D., Jha S.K., Durgesh K., Sathee L. (2022). Micro RNA Mediated Regulation of Nutrient Response in Plants: The Case of Nitrogen. Plant Physiol. Rep..

[30] Sharma P., Mishra S., Kaur A., Ahlawat O.P., Tiwari R. (2025). Novel and Conserved Drought-Responsive microRNAs Expression Analysis in Root Tissues of Wheat (*Triticum asetivum* L.) at Reproductive Stage. Front. Plant Sci..

[31] Sharaf A., Kundu J.K., Nuc P., Ibrahim E., Ripl J. (2025). Small Non-Coding RNAs in the Regulatory Network of Wheat Dwarf Virus-Infected Wheat. Agriculture.

[32] Ulu F., Unel N.M., Baloglu M.C. (2025). Genome-Wide Analysis of miRNAs and Their Target Genes in Wheat Cultivars with Different Ploidy Levels under Drought Stress. Planta.

[33] Hu Q., Liu H., He Y., Hao Y., Yan J., Liu S., Huang X., Yan Z., Zhang D., Ban X. (2024). Regulatory Mechanisms of Strigolactone Perception in Rice. Cell.

[34] Liu G., Liu F., Zhang D., Zhao T., Yang H., Jiang J., Li J., Zhang H., Xu X. (2023). Integrating Omics Reveals That miRNA-Guided Genetic Regulation on Plant Hormone Level and Defense Response Pathways Shape Resistance to Cladosporium Fulvum in the Tomato Cf-10-Gene-Carrying Line. Front. Genet..

[35] Li M., Ye X., Zhao Z., Zeng Y., Huang C., Ma X., Shuai P. (2025). Identification of miRNAs and Their Targets in Cunninghamia Lanceolata Under Low Phosphorus Stress Based on Small RNA and Degradome Sequencing. Int. J. Mol. Sci..

[36] Yu Y., Li W., Liu Y., Liu Y., Zhang Q., Ouyang Y., Ding W., Xue Y., Zou Y., Yan J. (2025). A Zea Genus-Specific Micropeptide Controls Kernel Dehydration in Maize. Cell.

[37] Wang Y., Zhang C., Hao Q., Sha A., Zhou R., Zhou X., Yuan L. (2013). Elucidation of miRNAs-Mediated Responses to Low Nitrogen Stress by Deep Sequencing of Two Soybean Genotypes. PLoS ONE.

[38] Li Q., Zhao X., Wu J., Shou H., Wang W. (2024). The F-Box Protein TaFBA1 Positively Regulates Drought Resistance and Yield Traits in Wheat. Plants.

[39] Wang Y., Shi C., Yang T., Zhao L., Chen J., Zhang N., Ren Y., Tang G., Cui D., Chen F. (2018). High-Throughput Sequencing Revealed That microRNAs Were Involved in the Development of Superior and Inferior Grains in Bread Wheat. Sci. Rep..

[40] Zhang H., Zhang X., Xiao J. (2023). Epigenetic Regulation of Nitrogen Signaling and Adaptation in Plants. Plants.

[41] Yang J., Duan G., Li C., Liu L., Han G., Zhang Y., Wang C. (2019). The Crosstalks Between Jasmonic Acid and Other Plant Hormone Signaling Highlight the Involvement of Jasmonic Acid as a Core Component in Plant Response to Biotic and Abiotic Stresses. Front. Plant Sci..

[42] Yuan T., Zhu C., Li G., Liu Y., Yang K., Li Z., Song X., Gao Z. (2022). An Integrated Regulatory Network of mRNAs, microRNAs, and lncRNAs Involved in Nitrogen Metabolism of Moso Bamboo. Front. Genet..

[43] Gao S., Yang Y., Yang Y., Zhang X., Su Y., Guo J., Que Y., Xu L. (2022). Identification of Low-Nitrogen-Related miRNAs and Their Target Genes in Sugarcane and the Role of miR156 in Nitrogen Assimilation. Int. J. Mol. Sci..

[44] Ma Y., Xue H., Zhang F., Jiang Q., Yang S., Yue P., Wang F., Zhang Y., Li L., He P. (2021). The miR156/SPL Module Regulates Apple Salt Stress Tolerance by Activating MdWRKY100 Expression. Plant Biotechnol. J..

[45] Gao S., Guo C., Zhang Y., Zhang F., Du X., Gu J., Xiao K. (2016). Wheat microRNA Member TaMIR444a Is Nitrogen Deprivation-Responsive and Involves Plant Adaptation to the Nitrogen-Starvation Stress. Plant Mol. Biol. Rep..

[46] Yang Y., Liang Y., Wang C., Wang Y. (2024). MicroRNAs as Potent Regulators in Nitrogen and Phosphorus Signaling Transduction and Their Applications. Stress Biol..

[47] Ali A., Jabeen N., Farruhbek R., Chachar Z., Laghari A.A., Chachar S., Ahmed N., Ahmed S., Yang Z. (2025). Enhancing Nitrogen Use Efficiency in Agriculture by Integrating Agronomic Practices and Genetic Advances. Front. Plant Sci..

[48] Zuo Z., Zhao H., Fan Y., Zhu Y., Song W., Zhai H., He S., Zhang H., Zhao N., Liu Q. (2025). Evolutionary Analysis of DELLA Proteins in Sweet Potato and Related Species Reveals Their Roles in Development and Stress Responses. Front. Plant Sci..

[49] Gaskin P., Kirkwood P.S., Lenton J.R., MacMillan J., Radley M.E. (1980). Identification of Gibberellins in Developing Wheat Grain. Agric. Biol. Chem..

[50] Zhou Y., Zhao C., Yan H., Yang J., Chen M., Wang X., Xie P., Ni Y., Niu J., Ren J. (2025). Genome-Wide Identification of 13 miR5200 Loci in Wheat and Investigation of Their Regulatory Roles Under Stress. Genes.

[51] Das S., Singh D., Meena H.S., Jha S.K., Kumari J., Chinnusamy V., Sathee L. (2023). Long Term Nitrogen Deficiency Alters Expression of miRNAs and Alters Nitrogen Metabolism and Root Architecture in Indian Dwarf Wheat (*Triticum sphaerococcum* Perc.) Genotypes. Sci. Rep..

[52] Sullivan L.M., Vierstra D.R. (1989). A Ubiquitin Carrier Protein from Wheat Germ Is Structurally and Functionally Similar to the Yeast DNA Repair Enzyme Encoded by RAD6. Proc. Natl. Acad. Sci. USA.

[53] Love M.I., Huber W., Anders S. (2014). Moderated Estimation of Fold Change and Dispersion for RNA-Seq Data with DESeq2. Genome Biol..

[54] Trick A.Y., Chen F.-E., Schares J.A., Freml B.E., Lor P., Yun Y., Wang T.-H. (2021). High Resolution Estimates of Relative Gene Abundance with Quantitative Ratiometric Regression PCR (qRR-PCR). Analyst.

[55] Gustavsson E.K., Zhang D., Reynolds R.H., Garcia-Ruiz S., Ryten M. (2022). Ggtranscript: An R Package for the Visualization and Interpretation of Transcript Isoforms Using Ggplot2. Bioinformatics.

