# Supplementary material for: miRNA Profiling Reveals the Role of Gibberellin Signaling Pathway in Low-Nitrogen Stress Adaptation of Xinjiang Spring Wheat
Source: Plants (Basel). 2026 Apr 2;15(7):1095. doi: 10.3390/plants15071095 (PMC13074741; doi:10.3390/plants15071095)
Supplement: Supplementary file 1 [file plants-15-01095-s001.zip › Supplementary Figures.pdf]

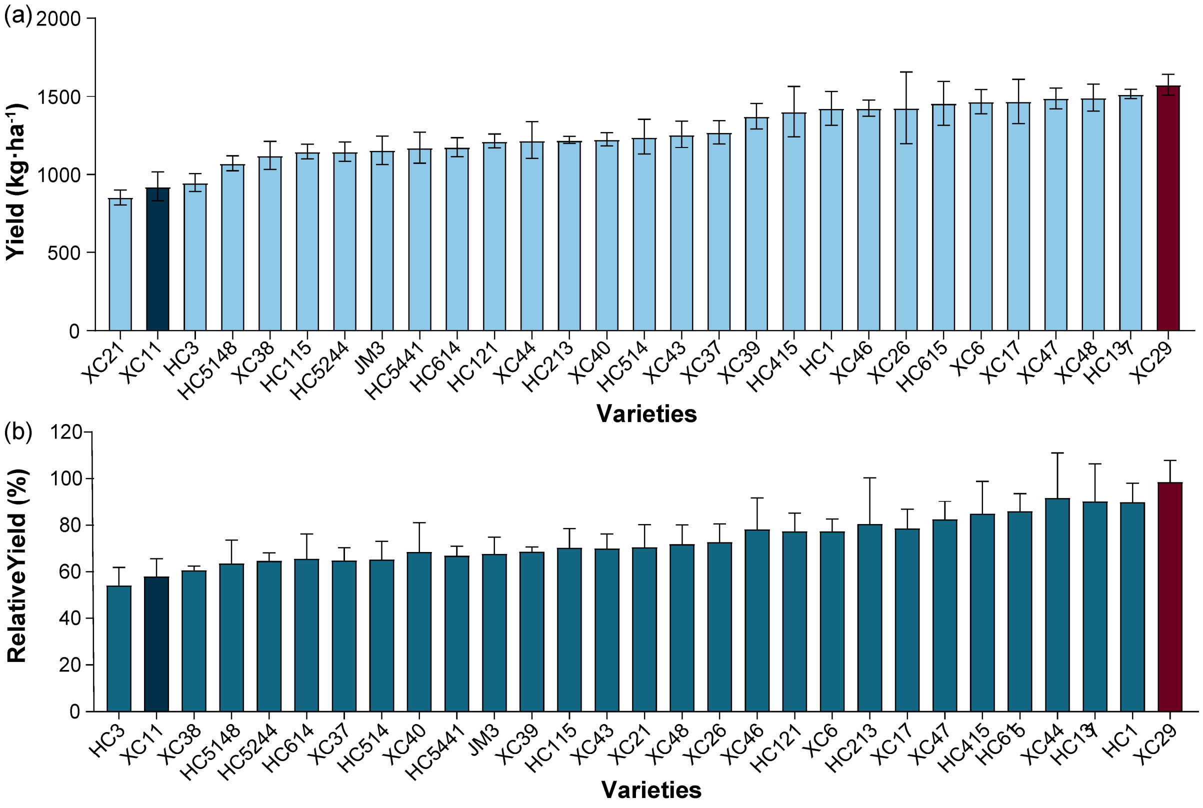

Figure S1. Supplementary Figure 1. Yield and relative yield nitrogen sensitivity of 29 cultivars under low nitrogen (LN) conditions

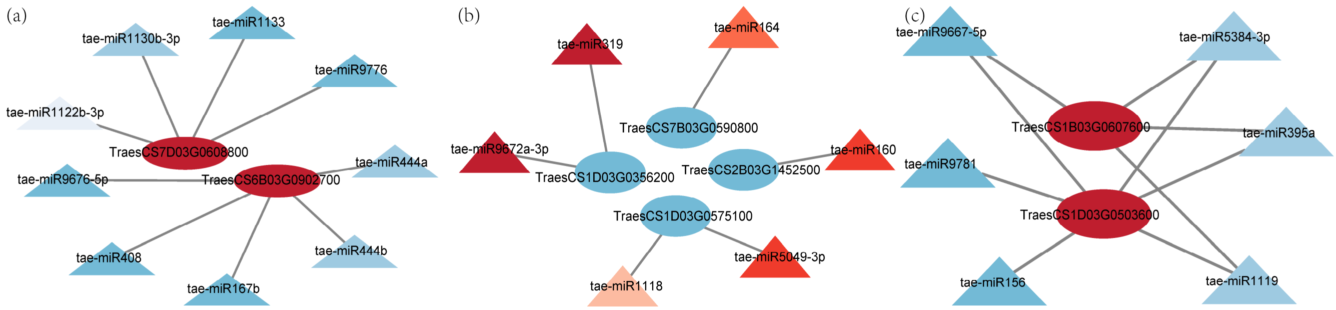

Figure S2. Selected results of expression correlation analysis between miRNAs and their targets.

(A) Positive regulatory effect of miRNAs with their target; (B) Negative regulatory effect of miRNAs with their target; (C) Regulatory effect of different miRNAs with their common target.

Note: Colors indicate the strength of expression correlation between miRNAs and their target genes.
Panels A and C represent positive regulation, while panel B represents negative regulation.
Darker colors indicate stronger expression correlation, and lighter colors indicate weaker correlation

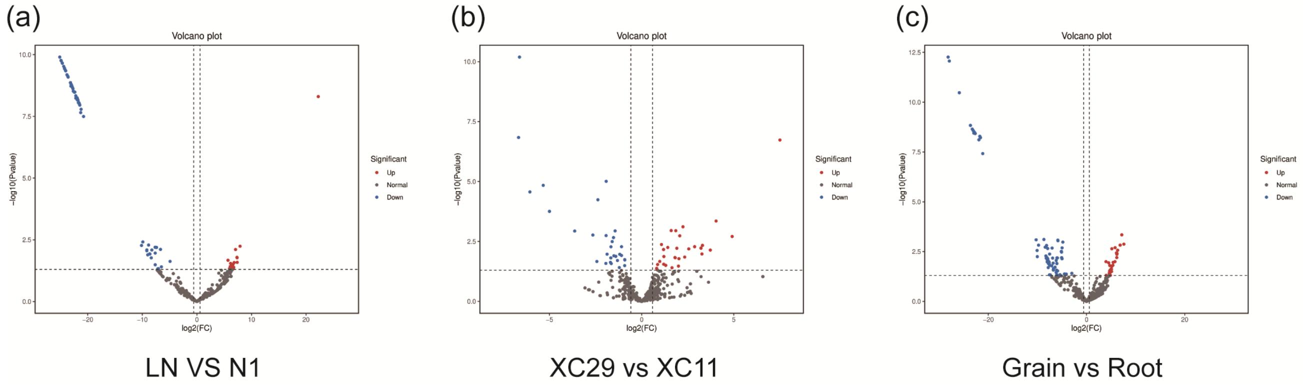

Figure S3. Volcano plot of differentially expressed miRNAs under low-nitrogen stress.

Note: $P < 0.05$; a total of 24 sample libraries were used.
